# Enhanced accuracy for classification of video capsule endoscopy images using multiple deep learning convolutional neural networks

**DOI:** 10.1016/j.igie.2023.11.007

**Published:** 2023-11-29

**Authors:** Dongguang Li, David Cave, April Li, Shaoguang Li

**Affiliations:** 1 Division of Hematology/Oncology, Department of Medicine, University of Massachusetts Chan Medical School, Worcester, Massachusetts, USA; 2 Brigham and Women’s Hospital, Boston, Massachusetts, USA; 3 Faculty of Medicine, The University of Queensland, Brisbane, Queensland, Australia

## Abstract

**Background and aims:**

Video capsule endoscopy (VCE) is widely used in the detection of abnormalities in the small intestine. However, it remains challenging to correctly identify a limited number of possible abnormal images from tens of thousands of total images, and this impediment has limited expansion of the technology. More recently, artificial intelligence (AI) technology has been used in classifying VCE images from patients, but clinical-grade diagnostic accuracy (>99%) has not been achieved.

**Methods:**

This study proposes a system for the automatic classiﬁcation of a number of categories of unbounded VCE images with high accuracy by means of a transfer learning approach using multiple convolutional neural networks (CNNs). With this new approach, it is not necessary to implement image segmentation; thus, the feature extraction becomes automatic, and the existing models can be ﬁne-tuned to obtain speciﬁc classiﬁers.

**Results:**

More than 16,000 VCE GI images from normal individuals, including those with normal clean mucosa, the pylorus, the ileocecal valve, a reduced mucosal view due to luminal contents and lymphangiectasia (a normal variant), and patients with 5 pathologic states (angioectasia, bleeding, erosions, ulcers, and foreign bodies), were obtained from a publicly available data set. These were used in building, testing, and validating AI models for evaluating the diagnostic accuracy of our combined 17-CNN deep learning approach. Compared with a single CNN approach used by other research groups, our AI method, using 17 CNNs, achieved an overall diagnostic accuracy of 99.79%, with an accuracy of 100% for identifying bleeding and foreign bodies. The high accuracy was further shown in the confusion matrices, precision, recall, and F1 score.

**Conclusions:**

We have developed accurate AI deep learning models for unbounded VCE image classification of various medical conditions in medical practice.

The human GI tract comprises the esophagus, stomach, small intestine, and colon, in which various disorders may occur, including, but not limited to, benign conditions such as bleeding, angioectasias, erosions, and ulcers, with the latter often associated with Crohn’s disease and neoplasia such as lymphoma and adenocarcinoma.^1^, ^2^, ^3^ Diagnosis of disease in the small intestine has been greatly facilitated by the advent of video capsule endoscopy (VCE).^4^

VCE has been available since 2001 for the detection of small intestinal disorders.^5^ VCEs are small, noninvasive, swallowable, and do not require sedation or anesthesia for deployment. The VCE captures live images as it is moved passively along by peristalsis (375-500 cm). This has created the challenge of how to correctly identify pathologic lesions represented on as little as 1 frame from tens of thousands of images. Even with the available limited image processing that eliminates repetitive images, the process can still take 20 to 60 minutes, with a significant error and incorrect interpretation rate. This labor-intensive challenge has played a substantial role in reducing the deployment of this technology.

Artificial intelligence (AI) has been used successfully in analyzing pathologic images for the diagnosis of human diseases with high accuracy,^6^^,^^7^ and computer-aided diagnostic tools, using deep learning, show promise in the performance of diagnostic classification. Deep learning is a type of machine learning in which a model learns to perform classification tasks directly from images and is usually implemented using neural network architecture.^8^ Transfer learning is an approach that applies knowledge of 1 type of problem to a different but related problem.^9^ Using a pretrained network with transfer learning is typically much faster and easier than training a network from scratch. Medical image analysis and computer-assisted intervention problems have been increasingly addressed with deep learning–based solutions.^10^ Available deep learning platforms are flexible, but they do not provide specific functionality for medical image analyses, and their adaption for this domain requires a substantial implementation effort.^11^ Consequently, there has been a considerable duplication of effort and incompatible infrastructure development between research groups.^12^

AI is predicted to have profound effects on the future of VCE technology,^13^^,^^14^ and recently, AI technology has been used in classifying capsule endoscopy images from patients.^15^ Although the results are promising in reducing the workload of gastroenterologists and improving diagnosis, a clinical-grade diagnostic accuracy (>99%) has not been achieved by any research group. In the current study, we analyzed unbounded VCE images from a publicly available data set (Kvasir-Capsule)^16^ and generated accurate AI deep learning models for VCE image classification of various medical conditions in the small intestine.

## Methods

### VCE images

All images were obtained in the color space RGB.

### Convolutional neural networks

Instead of using a single model, we introduced a collection of 17 models to establish our system, including AlexNet, GoogleNet, ResNet18, SqueezeNet, MobileNetv2, Inceptionv3, DenseNet201, Xception, Places365GoogleNet, InceptionResNetv2, ResNet50, ResNet101, NASNetMobile, ShuffleNet, DarkNet19, DarkNet53, and EfficientNetb0. These CNN deep learning models had been trained on approximately 1.2 million images from the ImageNet data set^17^ or a subset of the ImageNet database, with an ability to classify images into 1000 object categories.

To get multiple models to work together, the input image size must be resized to meet the various requirements of those models before conducting training and testing. Table 1 shows a list of input image sizes for each pretrained model involved in this study.

**Table 1:** The input image sizes of pretrained networks

No.	Name of the pretrained networks	Input image size
1	GoogleNet	224 × 224
2	Vgg16	224 ×224
3	Vgg19	224 × 224
4	ResNet18	224 × 224
5	ResNet50	224 × 224
6	ResNet101	224 × 224
7	MobileNetv2	224 × 224
8	DenseNet201	224 × 224
9	Places365GoogleNet	224 × 224
10	NASNetMobile	224 × 224
11	ShuffleNet	224 × 224
12	EfficientNetb0	224 × 224
13	AlexNet	227 × 227
14	SqueezeNet	227 × 227
15	DarkNet19	256 × 256
16	DarkNet53	256 × 256
17	Inceptionv3	299 × 299
18	Xception	299 × 299
19	InceptionResNetv2	299 × 299
20	NASNetLarge	331 × 331

To classify a single image, the final classified category is decided by majority rule.^18^ The majority rule is a decision rule that selects alternatives that have a majority; that is, maximum votes among those models involved. This idea has been introduced in this study from 1 of the election theories called approval voting. Under approval voting, a voter indicates which candidates he or she approves. A candidate receives 1 point for each voter that approves of the candidate. A candidate receives no points for each voter that does not approve of the candidate. For a single candidate election, the candidate with the most points wins the election. Naturally, approval by all candidates or disapproval by all candidates does not change the difference in the number of points the candidates receive. If there is an odd number of voters and no voter approves or disapproves of both candidates, the approval voting is equivalent to majority rule: each voter gives 1 point to the candidate that he or she prefers, and the candidate with a majority of the points wins the election.

Determining a winner for a 2-candidate election is easy, as it is a binary classification problem. It has been shown that majority rule is the only 2-candidate election procedure in which each voter is treated equally.^19^ Only the number of votes matters, not who casts the votes. Each candidate is treated equally; that is, only the number of votes that a candidate receives determines if they win the election, and a candidate can never be harmed by receiving more votes (ie, if a candidate wins the election, they would still win the election if some of the voters who had voted for the candidate’s opponent now voted for the candidate). Elections with 3 or more candidates, which is the case in this study with 10 capsule endoscopy image category candidates, can also easily be simulated based on the voting mechanism.

For the multiple CNN models to work together, a core algorithm was developed based on the aforementioned voting mechanism. In the classification process, each individual CNN model votes for 1 of the image categories, and the category that has been voted for will score 1 point. A 2-dimensional array vote (scores[i], categories[i]) is created after receiving all of the CNNs’ contributing vote scores. Then the final classification output, the category, can be calculated by adding up all scores of the multiple CNNs, and the category that has a maximum score is the winner.

(Equation 1) A=rank(Vote[scores(i),categories(i)];i=1,2,…numberofcategories

(Equation 2) Classifiedcategory=A(categories[1])

The definition of the accuracy over the testing data set is given as:

(Equation 3) Accuracy=(TC/[TC+FC])×100

True classified (TC) indicates the number of images correctly classified. False classified (FC) indicates the number of images incorrectly classified. In other words, the accuracy of a test is defined as its ability to differentiate the image categories correctly.

The average of all 17 single CNNs involved in this study can be calculated as follows:

(Equation 4) $$\begin{array}{l} A=\sum_{k=1}^{17}a\left(k\right)\\ \text{Average}\;\text{accuracy}=A/17 \end{array}$$

Diagnostic accuracy was not the only measure for evaluating diagnostic performance in this study. To further evaluate our deep learning platform, we used additional evaluation measures, which involved the use of the following terms: true positive (TP), false positive, true negative, and false negative (FN).

(Equation 5) Precision(positivepredictivevalue)=TP/(TP+falsepositive)

(Equation 6) Recall(sensitivity)=TP/(TP+FN)

(Equation 7) F1score=2×[(precision×recall)/(precision+recall)]

### Hardware and software

The MATLAB Deep Learning Toolbox (MathWorks, Natick, Mass, USA) provides a framework for designing and implementing deep neural networks with algorithms, pretrained models, and apps. The Image Processing Toolbox provides a comprehensive set of reference standard algorithms and workflow apps for image processing, analysis, visualization, and algorithm development. The networks in this study were all trained and tested by using MATLAB software (MATLAB R2021a) and a Titan XP GPU (NVIDIA, Santa Clara, Calif, USA).

### Code availability

The source code, including the deep learning models developed in this study, can be downloaded from our FTP site (https://fts.umassmed.edu/ [user name: lid1; password: April2000april2000]) in the folder of /Home/lid1/Capsule Endoscopy Code and Data.

### Data availability

The data sets used in this study can be downloaded from our FTP site (https://fts.umassmed.edu/ [user name: lid1; password: April2000april2000]) in the folder of /Home/lid1/Capsule Endoscopy Code and Data.

## Results

### Data set

**Figure 1:**
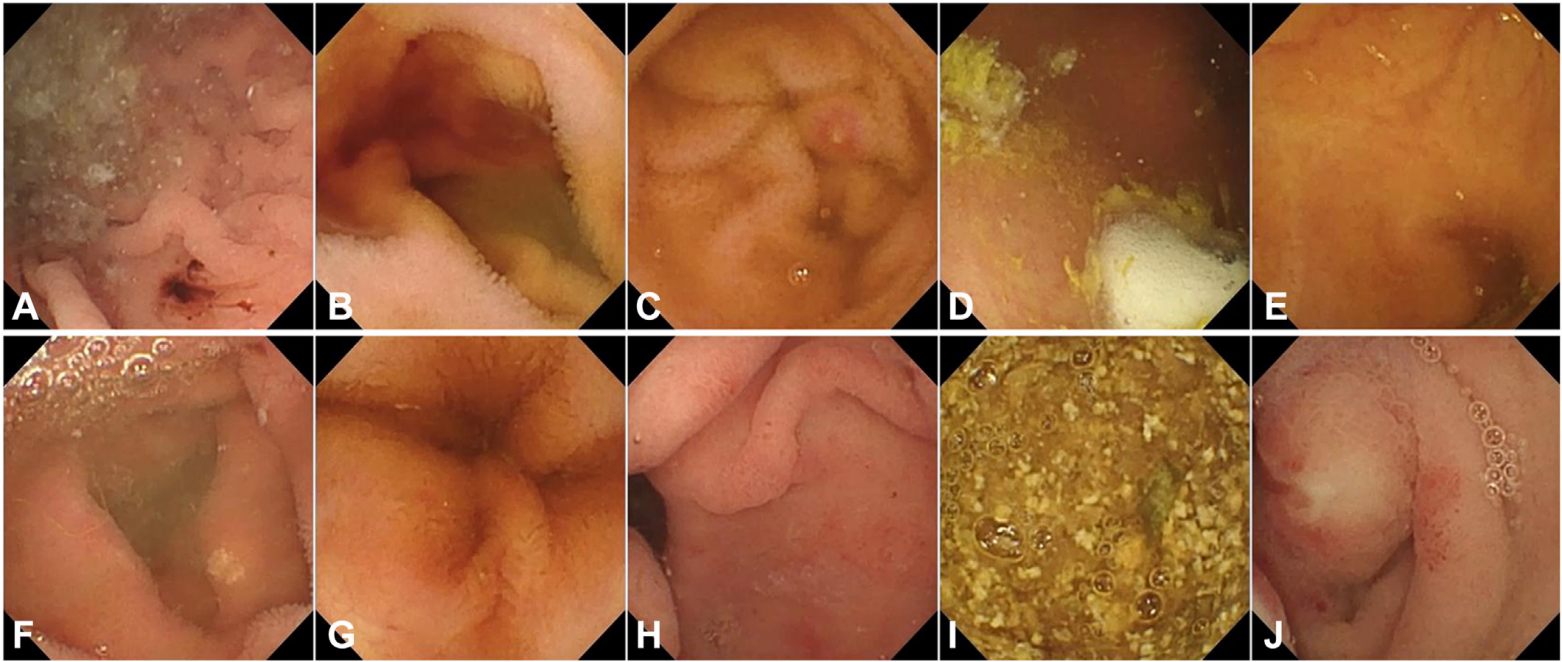
Samples of images acquired by the capsule endoscope. Ten types of images acquired by the capsule endoscopy are shown. **A,** Angiectasia. **B,** Fresh blood. **C,** Erosion. **D,** Foreign body. **E,** Ileocecal valve. **F,** Lymphangiectasia. **G,** Normal clean mucosa. **H,** Pylorus. **I,** Reduced mucosal view. **J,** Ulcer.

The Kvasir-Capsule data set is available from the Open Science Framework.^20^ Originally, the videos are captured at a variable frame rate of 3 to 5 frames per second, at a resolution of 336 × 336 pixels using the Olympus EC-10 capsule to acquire the images. In total, the data set consists of 4,820,857 main data records and 44,228 images with labels. From the Kvasir-Capsule data set, we extracted as much data as possible for 10 categories of small intestinal images for them to be appropriate for all deep learning techniques (Table 2). The data set contains a total of 16,124 individual capsule endoscopy images that are organized in the following 10 groups: normal clean mucosa, reduced mucosal view, pylorus, ileocecal valve, and lymphangiectasia (normal variant); and images containing pathologic abnormalities such as angioectasias, fresh blood, erosions, ulcers, and foreign bodies (Table 2; Fig. 1).

**Table 2:** Overview of the image distribution in the data set

Image type	Total no. of images by type	%
Angiectasia	868	5.38
Fresh blood	452	2.80
Erosion	506	3.14
Foreign bodies	776	4.81
Ileocecal valve	4189	25.98
Lymphangiectasia	592	3.67
Normal clean mucosa	3462	21.47
Pylorus	1529	9.48
Reduced mucosal view	2906	18.02
Ulcer	844	5.23
Total	16,124	100

### Strategies for building AI models

**Figure 2:**
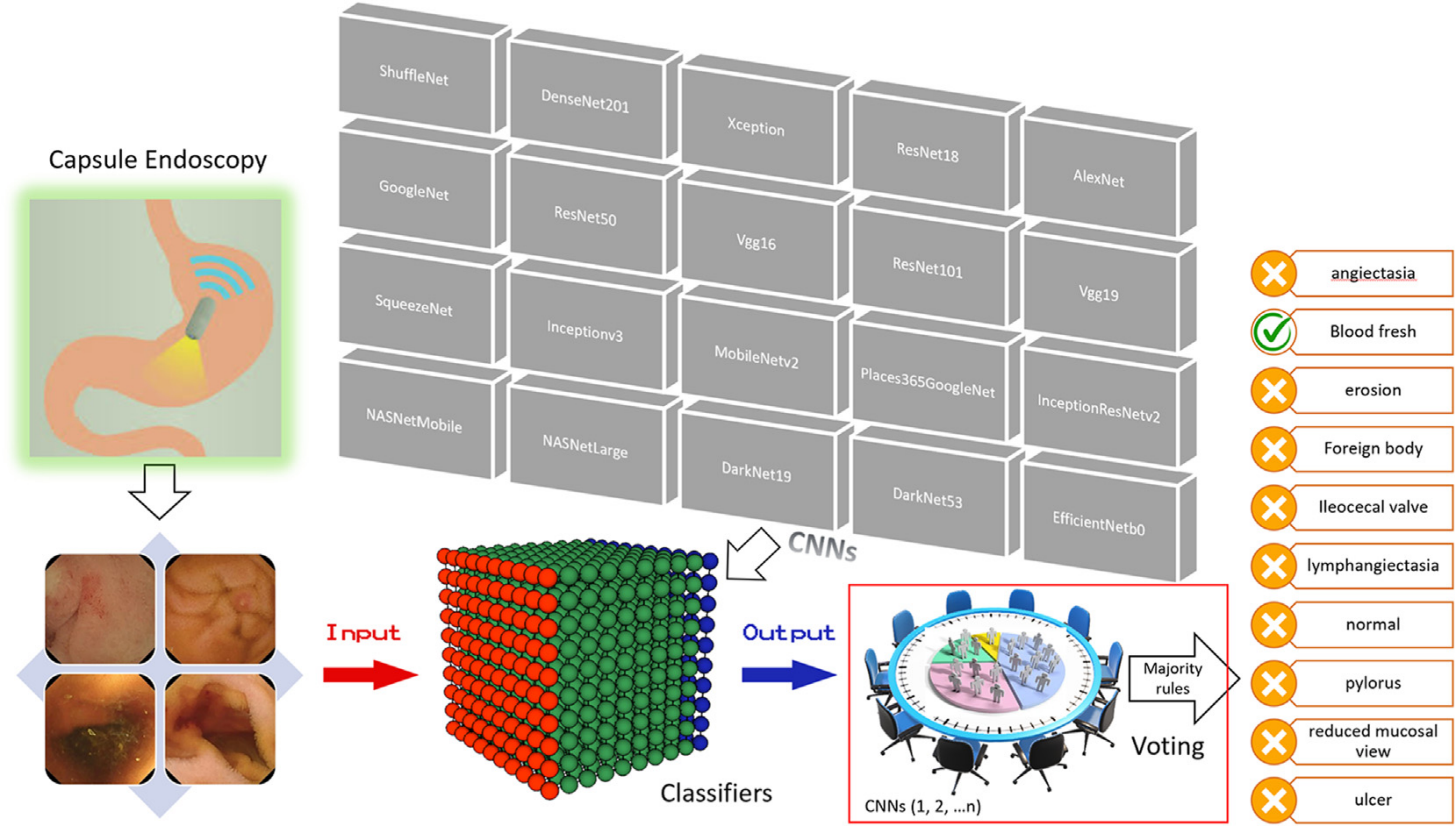
An illustration of our artificial intelligence deep learning approach in capsule endoscopy image classification. Unbounded video capsule endoscopy images were used for analysis by artificial intelligence deep learning. *CNN*, Convolutional neural network.

**Figure 3:**
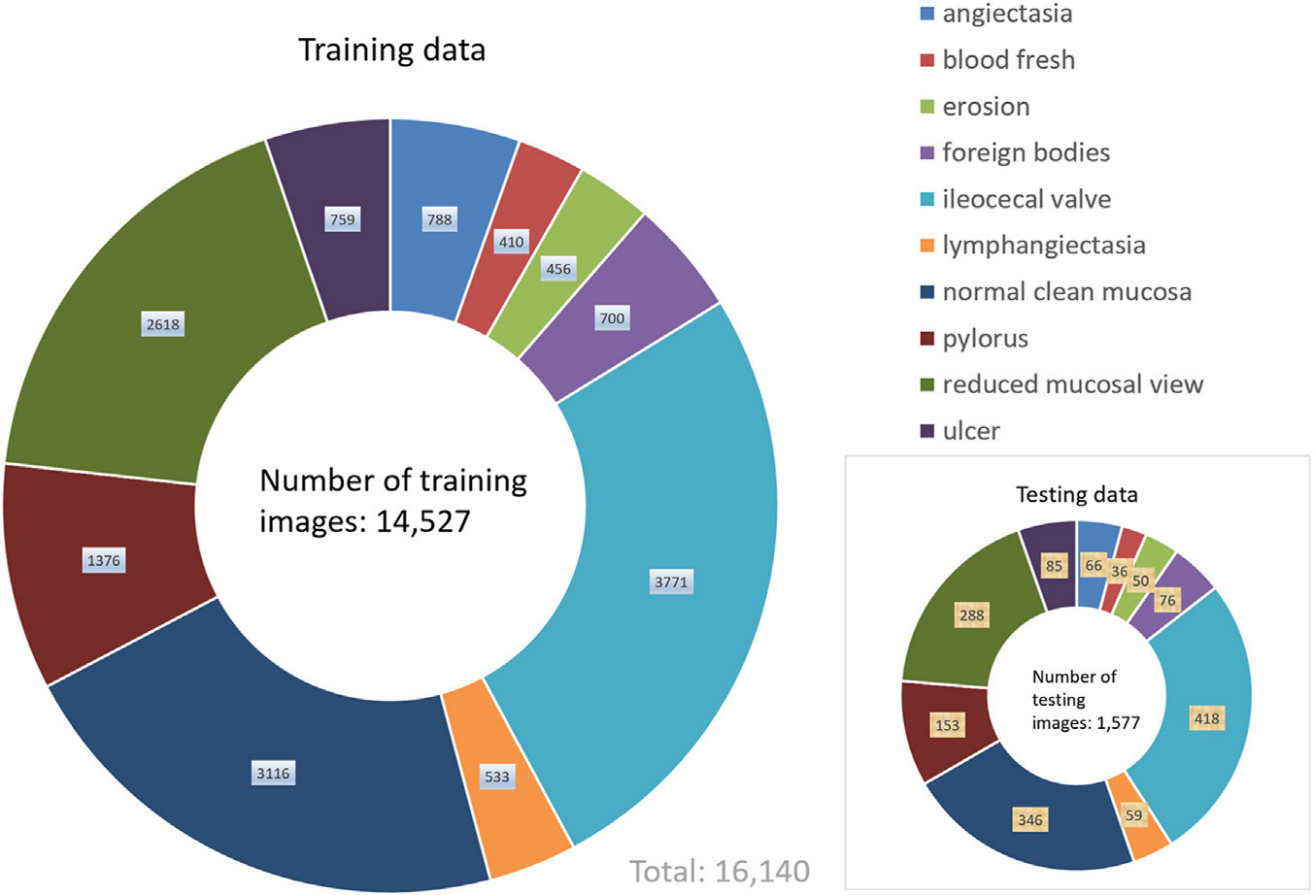
Data distribution between training and testing among 10 types of video capsule endoscopy images.

Unlike a single CNN model approach commonly used by other researchers, we combined multiple CNNs and used them jointly as a single model in building AI models. This single model has all layers built in those multiple CNNs with an expectation for conducting transfer deep learning with any complex data sets to achieve higher classification accuracy for the 10 types of VCE images analyzed in this study (Fig. 2); this could not have been achieved by using a single CNN model approach. The data set for each type of VCE images was randomly divided into a training data set and a testing data set, with approximately 90% of the images for training an AI model and 10% of the images put aside as unseen data for testing the model (Fig. 3).

### Achievement of high classification accuracy

**Figure 4:**
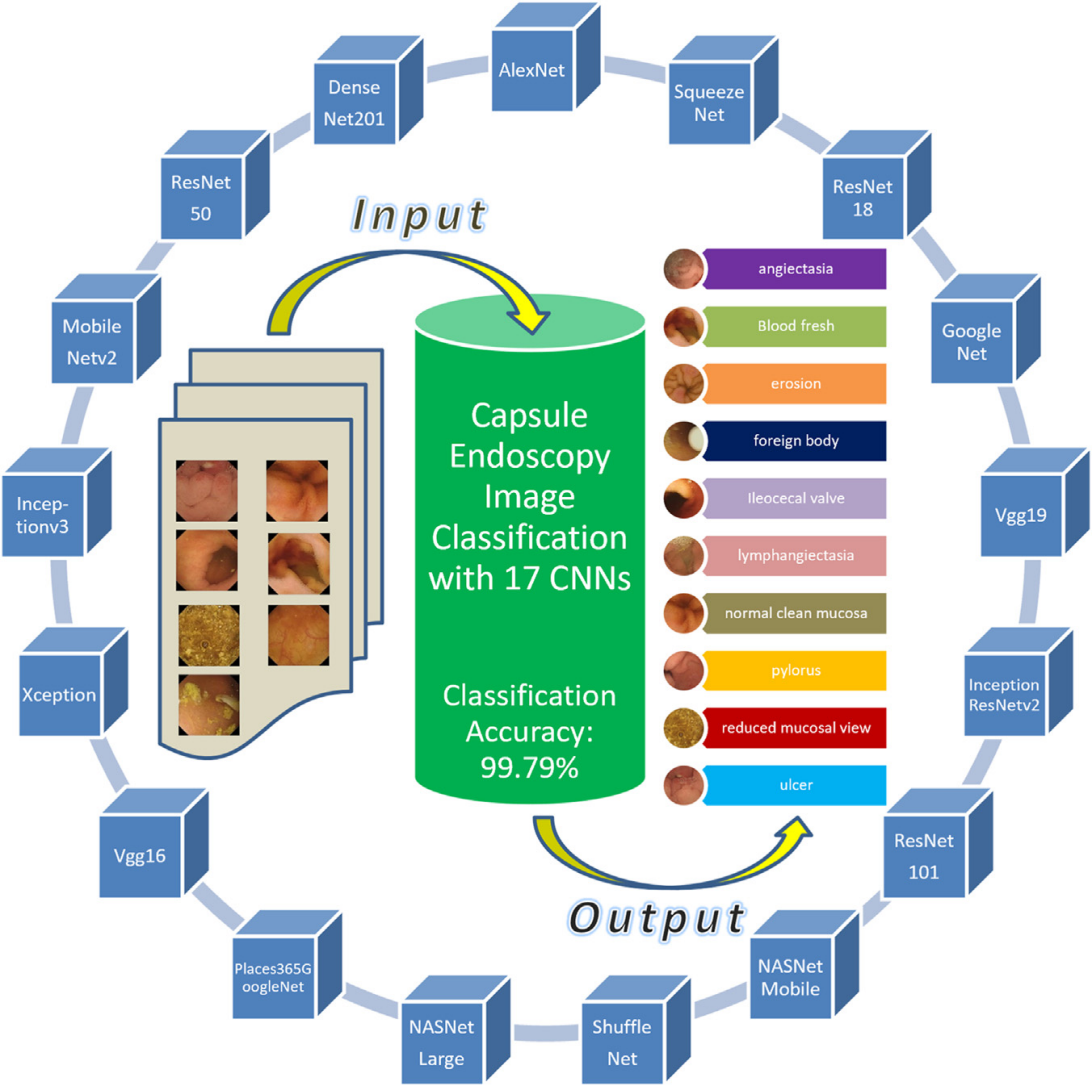
Classification of video capsule endoscopy images using a multiple convolutional neural network (CNN) approach. A high overall classification accuracy has been achieved.

**Figure 5:**
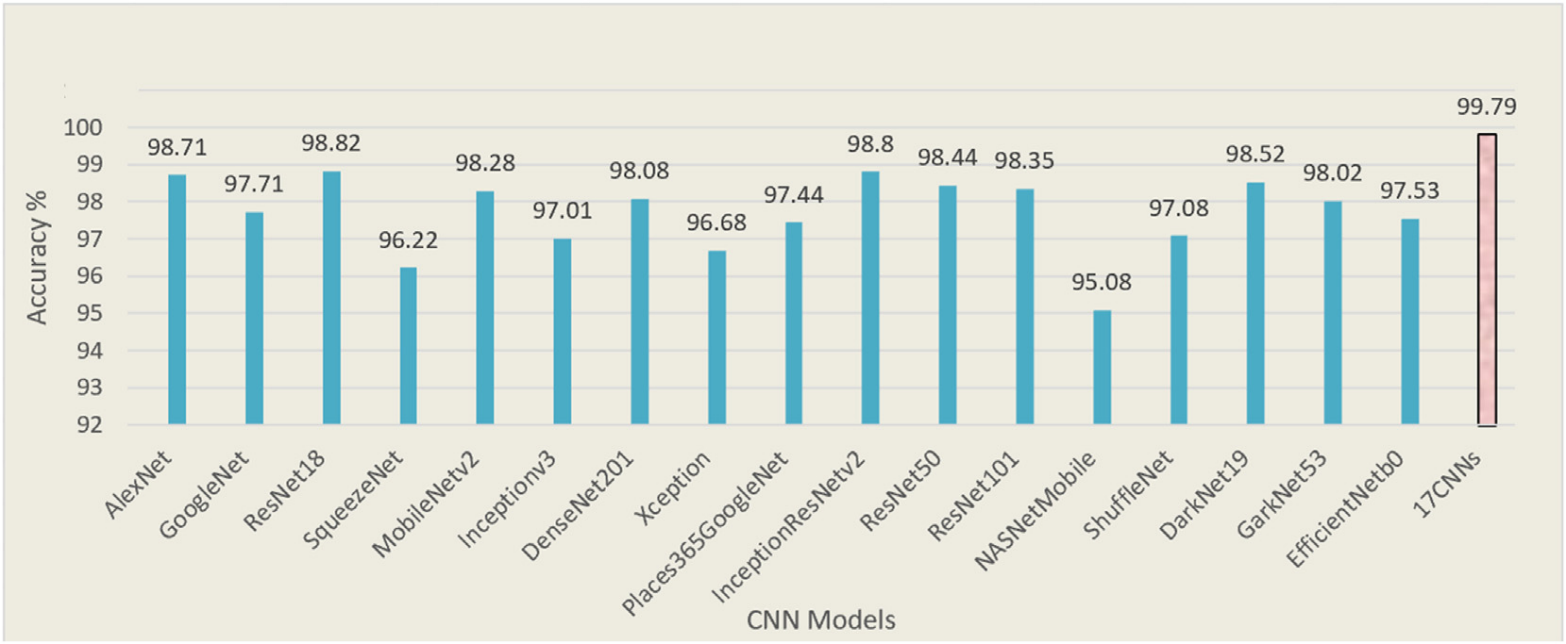
Comparison of classification accuracies with a single convolutional neural network (CNN) approach. The multiple CNN approach allows for achieving the highest classification accuracy compared with the single CNN approach.

**Figure 6:**
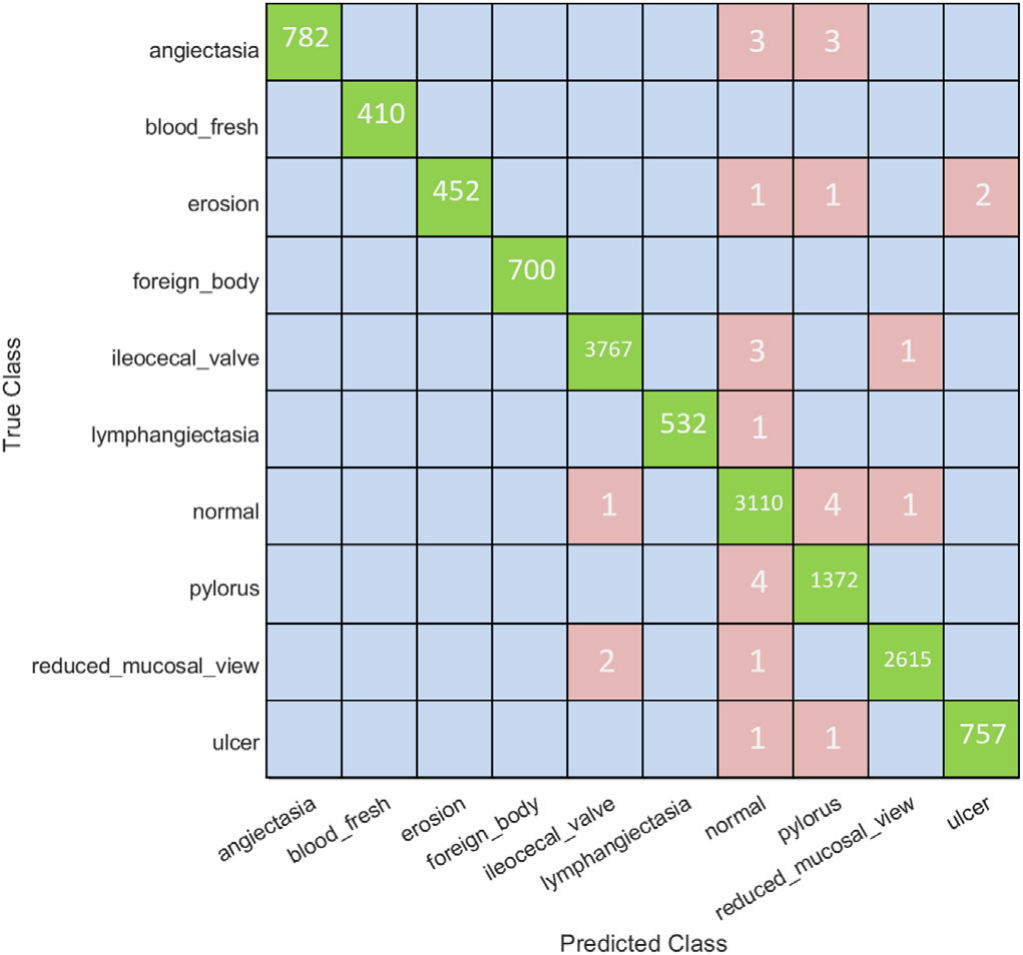
Confusion matrix based on the training image data set. Our multiple convolutional neural network model shows enhanced performance in classification for multiple subtypes of images.

**Figure 7:**
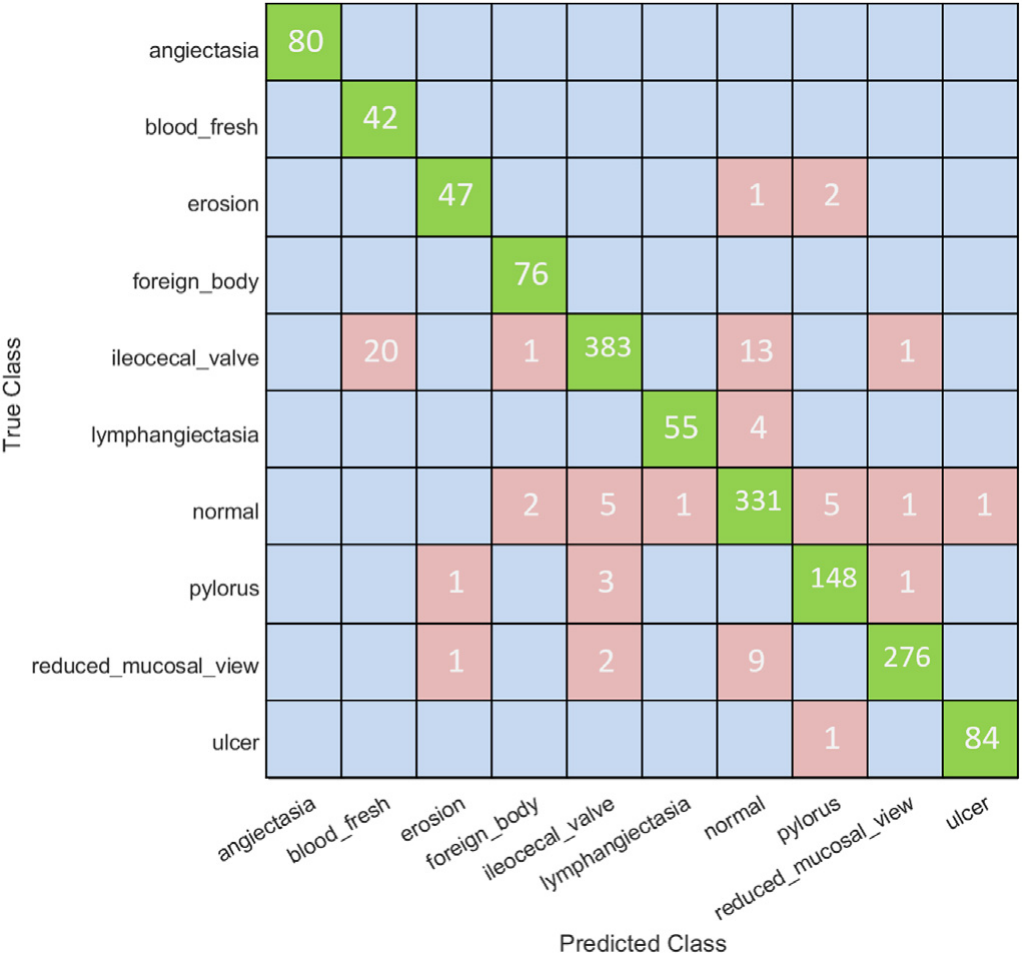
Confusion matrix based on the testing image data set. Our multiple convolutional neural network model performs well on the testing image data set.

Classification accuracy was used to evaluate the performance of all classifiers. As described earlier (Fig. 3), the data set for each classifier was randomly split into training (90% images) and testing (10% images) data sets. Seventeen pretrained models (Table 2) were used in constructing a multiple CNN system to train our AI models on the training data set (Fig. 4). We used 90% of each type of VCE image to train or establish an AI model and the remaining 10% of the images, which were unseen during the establishment of the AI model, for testing the model to evaluate the classification accuracy. As a result, for the training data set, our AI models with the combined 17 CNNs allowed achievement of an overall classification accuracy of 99.79%. This high classification accuracy was better than any overall accuracy achieved by using a single CNN (Fig. 5). The overall accuracy achieved by each single CNN varied between 95.08% and 98.82%, which was lower than the accuracy achieved by our combined 17-CNN approach. Moreover, the high accuracy achieved via our combined 17-CNN approach was further shown by the classification outcomes shown in the confusion matrices for both training (Fig. 6) and testing (Fig. 7) data sets.

We were also curious about the performance of our combined 17-CNN approach on classification of each type of the VCE images. We found that 100% classification accuracy was reached for identifying fresh blood and foreign bodies, and the accuracies for the 8 other types of images were >99% (Table 3). To further validate our AI models, we tested the remaining 10% of each type of the VCE images that were unseen during establishment of the AI models and achieved an overall classification accuracy of 95.30%, with 100% accuracy for fresh blood and foreign bodies (Table 4). It should be noted that each CNN used in the study had been trained with optimized algorithms through transfer learning on the same training data set and was tested on the corresponding testing data set.

**Table 3:** The perfomance of our classifier with 17 combined CNNs

Image type	No. of images in total	Classified correctly	Classified incorrectly	Classification accuracy, %	Precision, %	Recall, %	F1 score, %
Angioectasia	788	782	6	99.24	99.24	100	99.62
Blood fresh	410	410	0	100	100	100	100
Erosion	456	452	4	99.12	99.12	100	99.56
Foreign bodies	700	700	0	100	100	100	100
Ileocecal valve	3771	3767	4	99.89	99.89	99.92	99.90
Lymphangiectasia	533	532	1	99.81	99.81	100	99.90
Normal clean mucosa	3116	3110	6	99.81	99.81	99.55	99.68
Pylorus	1376	1372	4	99.85	99.71	99.35	99.53
Reduced mucosal view	2618	2615	3	99.89	99.89	99.92	99.90
Ulcer	759	757	2	99.74	99.74	99.74	99.74
All images	14,527	14,497	30	99.79			99.79

*CNNs*, Convolutional neural networks.

**Table 4:** The perfomance of our combined 17-CNN approach on the testing data set

Image type	No. of images in total	Classified correctly	Classified incorrectly	Classification accuracy, %	Precision, %	Recall, %	F1 score, %
Angioectasia	80	80	0	100	100	100	100
Fresh blood	42	42	0	100	100	67.74	80.77
Erosion	50	47	3	94.0	94.0	95.92	94.95
Foreign bodies	76	76	0	100	100	96.20	98.06
Ileocecal valve	418	383	35	91.63	91.63	97.46	94.46
lymphangiectasia	59	55	4	93.22	93.22	98.21	95.65
Normal clean mucosa	346	331	15	95.66	95.66	92.46	94.02
Pylorus	153	148	5	96.73	96.73	94.87	95.79
Reduced mucosal view	288	276	12	95.83	95.83	98.92	97.35
Ulcer	85	84	1	98.82	98.82	98.82	98.82
All images	1597	1522	75	95.30			95.39

*CNNs*, Convolutional neural networks.

Additional evaluation measures, including precision (positive predictive value), recall (sensitivity), and F1 score, were used to further evaluate the deep learning platform; this platform allowed us to obtain high values in all of these evaluation measures (Tables 3 and 4). When calculating the overall F1 score, we did not give equal weights to each class. Instead, a weighted-average F1 score was introduced in which the F1 score of each class was weighted by the number of samples from that class. More significantly, the weighted F1 scores can be calculated for both the training data set and the testing data set, which are 99.79% and 95.39%, respectively.

## Discussion

In our previous studies on medical image classification using multiple CNNs,^6^^,^^7^ we introduced an innovative concept and a methodology that combines multiple CNNs in a way that can be reliably used to increase medical image classification accuracy using comparatively limited resources. Our AI method allows for building deep learning models using smaller data sets, making it possible to employ our AI method in medical practice. Moreover, in practice, it would be difficult to know or choose an appropriate single CNN without testing all CNNs individually, which is time-consuming and expensive. In contrast, our combined 17-CNN approach ensures the building of AI models that achieve the highest classification accuracy. In other words, our combined 17-CNN approach ensures the best performance in analyzing VCE images compared with the use of any single CNN.

We chose 17 CNNs in this study for 2 reasons. First, we intended to run all CNNs using a regular laptop or desktop computer that is economical and easy to use, and the GPU hardware only allows running some available CNNs efficiently. Second, the memory capacity required for each CNN is different, limiting the use of some CNNs in our study for high efficiency. In our experience, at least 10 CNNs should be used in the multiple CNN approach to achieve the best results. Conversely, although the time for training the 17-CNN deep learning model is longer than that for training the single CNN model (taking only hours or days at most), which is reasonable and manageable, the time for running the 17-CNN deep learning model is similar to that for running the single CNN model (taking a few seconds), which causes no delay in the analysis of VCE images in medical practice. Usually, training a deep CNN from scratch is time-consuming. However, this issue can be resolved by leveraging existing CNNs that have been trained on large data sets for conceptually similar tasks, a process called transfer learning. In establishing our 17-CNN deep learning model, we applied the transfer learning mechanism, which allowed for a quicker building of the 17-CNN deep learning approach.

We should note that accuracy only measures the number of correctly predicted values among the total predicted value. Although it is a good measure of performance, it is not complete and does not work well when the cost of false-negative findings is high. This is why we used more evaluation measures (including precision, recall, and F1 score) and our deep learning platform to show that we obtained high values in all these evaluation measures. In addition, we chose to study the 10 categories of disease and healthy classifications marked in the HyperKvasir data set, but this does not mean that we were only interested in studying those 10 categories. We are capable of studying any type of GI diseases, as long as we have access to a large enough number of such cases, which is a general requirement for conducting AI-related studies. Thus, our plan is to study all categories of GI diseases after being able to collect enough cases. However, we realize that some diseases, such as small intestinal neoplasia, are of high clinical significance, but it is difficult to obtain a large number of cases in a timely manner.

In this study of VCE image classification, we have shown the capabilities of this methodology to achieve high diagnostic accuracy for various medical conditions in the small bowel by analyzing a smaller number of VCE images compared with similar studies conducted by other research groups. It is notable that our methodology allows for achieving an overall diagnostic accuracy of 99.79% for various common disorders in the small bowel and 100% accuracy for bleeding and foreign bodies. Specifically, the accuracy of our method, from a relatively small number of images, seems to be comparable to the very large number of images reviewed in a recent study, in which a CNN auxiliary model was used to analyze images collected from 6970 patients in 77 sites using the Ankon video capsule (Ankon Technologies, Shanghai, China).^21^ A total of 158,235 images were culled from 1970 patients to create a training set; their model was validated in 5000 patients unrelated to the validation set, achieving a sensitivity of 99.88% in their per-patient analysis compared with 74.57% sensitivity achieved by a panel of expert gastroenterologists. Reading time by gastroenterologists was 96.66 ± 22.53 minutes compared with 5.9 ± 2.23 minutes with the CNN-based system. A similar-scale study was reported using the OMOM capsule (Jinshan Science and Technology, Chongqing, China) and a deep learning network (SmartScan), the YOLO network,^22^ in which the reading time was reduced from 51.4 minutes for gastroenterologists to 5.4 minutes. It is necessary to point out that compared with our study focusing on the performance in diagnostic accuracy, those studies emphasized the sensitivity of their methods with a concern of introducing a significant level of false positivity in identifying particular images, which would, at a certain degree, compromise the effort of reducing the workload by gastroenterologists using the AI technology. Also, the use of diagnostic accuracy is more helpful in medical practice, although the maintenance of a high sensitivity in detecting pathologic lesions is important. Moreover, the potential of the multiplex approach lies in improving anomaly detection while reducing manual labor. This is important because medical image data are often sparse or not easily available to the research community, and medical personnel rarely have time for the tedious labeling work involved in categorizing images.^15^

Going forward, the clinical application of machine learning (CNNs) to the detection of abnormalities in the small intestine by VCE not only requires image identification accuracy but also reasonably fast processing time. The need for high-speed real-time processing as is required for polyp detection during colonoscopy is not essential, but a processing time of a few minutes is necessary. One of the rate-limiting steps, presently, is the download time for the video from the capsule to the workstation, which may take from a few minutes to 1 hour more depending on the configuration of the download process. Previous studies have reported very substantial reductions in reading time with machine learning compared with human counterparts. The precise presentation of image analysis by the machine learning is usually not reported. There needs to be a clear description of how the selected images are presented to the clinician. This might include the presentation of a single image within a video or a segment of abnormality that can be easily and quickly reviewed and transferred to a report, which in turn can be distributed electronically to the electronic medical record.

For the foreseeable future, one of the limits of AI software, apart from the high cost, will be the need for physician review of the images for confirmation of any abnormalities and the subsequent generation of a report to provide an accurate diagnosis. In gastroenterology, the lack of specialists, heavy workload, inexperience, and a resulting wrong diagnosis can affect downstream management and treatment decisions, leading to incorrect treatment with an increase in associated costs and legal implications.

AI is dependent on validation on a specific device. Each device and its successor have variations in pixel number, image collection, and image processing among many other factors. The Kvasir-Capsule image set is derived from the Olympus EC-10,^16^ which unfortunately is no longer in production and so cannot be expanded. However, it remains a useful tool for developing new AI methods. The Ankon capsule and AI are available in China and Europe and pending U.S. Food and Drug Administration approval in the United States. The SB3 capsule (Medtronic, Minneapolis, Minn, USA) with AI is in development. A large image database using images from this capsule was created by a European consortium,^23^ but it is not clear if this is in the public domain.

We would like to point out that the individual still images we used were derived from videos. In a clinical VCE setting, video is the optimal input format for evaluation and analysis by clinicians. For the analysis of a video by any algorithm, the initial step that must be taken is to convert the source video to individual still images behind the user interface. When a video is reviewed for diagnosis of a particular disease, the examiner scans the video to focus on a single or small number of still images to refine their observation. Those “thumbnails” are then copied to a report. Thus, we focused on the still images in our study because this parallels the process ongoing in medical practice. On the other hand, algorithms tend to run very differently with still images versus videos. In the current study, we solved this problem by having a user-friendly interface based on Microsoft Windows (Microsoft Corporation, Redmond, Wash, USA) run our algorithms. Therefore, either videos or a collection of still images can be dealt with in our study as input to the system for conducting further diagnoses in the same way.

There are potential limitations in this study. We primarily used a pre-existing database generated in Norway. We were therefore dependent on a panel of gastroenterologists who had their own criteria for each set of normal and abnormal images, which may differ from other data sets. Differences in terminology can occur and are reduced by the use of the capsule endoscopy Minimum Standard Terminology (MST). The MST could be updated by an international panel of experts that could be convened to help provide a new criterion standard. A set of images for each condition (5-10) could be reviewed and a consensus generated, which could help refine the MST. However, the image database provides reliable data for testing the ability of our AI technology that can be used for analyzing other databases available in different hospitals. In this regard, we must note that variations of the images from different sources are often significant, relating to the types of the imaging machine/device, settings of the imaging machine/device, and procedural differences in collecting the images. It will be critical to standardize the method for collecting VCE images across hospitals. Realistically, the standardization requires huge efforts from the entire scientific community, and we plan to use another strategy. When we use an AI model built by training VCE images obtained from one source to analyze external, unfamiliar images from another source, we could combine a smaller set of the unfamiliar images into our prior training data set to create a new training data set and then re-train an AI model by reading the combined images. As a result, the new AI model will be applicable to analyzing future external VCE images that are unfamiliar to the new AI model built from reading the combined images from both sources.

In conclusion, we report a novel approach to the use of multiple CNNs that results in enhanced accuracy for detection of lesions in the GI tract with a significantly smaller data set. In addition, our AI deep learning approach eliminates the requirement for image bounding by medical experts, a time-consuming and labor-intensive process.

## Disclosure

All authors disclosed no financial relationships. S. Li: supported by grants from the National Institutes of Health (R01CA176179 and R01CA222590).
